# A New Orthodontic-Surgical Approach to Mandibular Retrognathia

**DOI:** 10.3390/bioengineering8110180

**Published:** 2021-11-08

**Authors:** Francisco Vale, Joana Queiroga, Flávia Pereira, Madalena Ribeiro, Filipa Marques, Raquel Travassos, Catarina Nunes, Anabela Baptista Paula, Inês Francisco

**Affiliations:** 1 Institute of Orthodontics, Faculty of Medicine, University of Coimbra, 3000-075 Coimbra, Portugal; joana1923@gmail.com (J.Q.); fppereira_@hotmail.com (F.P.); madalenaprata@hotmail.com (M.R.); filipa.p.s.marques@gmail.com (F.M.); raqueltravassos.91@gmail.com (R.T.); mcal9497@hotmail.com (C.N.); anabelabppaula@sapo.pt (A.B.P.); ines70.francisco@gmail.com (I.F.); 2 Institute of Integrated Clinical Practice, Faculty of Medicine, University of Coimbra, 3000-075 Coimbra, Portugal; 3 Coimbra Institute for Clinical and Biomedical Research (iCBR), Area of Environment Genetics and Oncobiology (CIMAGO), Faculty of Medicine, University of Coimbra, 3000-075 Coimbra, Portugal; 4 Centre for Innovative Biomedicine and Biotechnology (CIBB), University of Coimbra, 3000-075 Coimbra, Portugal; 5 Clinical Academic Center of Coimbra (CACC), 3000-075 Coimbra, Portugal

**Keywords:** distraction osteogenesis, mandible, orthognathic surgery

## Abstract

(1) Background: Mandibular deficiency is one of the most common growth disorders of the facial skeleton. Recently, distraction osteogenesis has been suggested as the treatment of choice for overcoming the limitations of conventional orthognathic surgery; (2) Methods: A new custom-manufactured dental-anchored distractor was built and anchored in the first molar and lower canine. It consists of a stainless-steel disjunction screw, adapted and welded to the orthodontic bands through two 1.2 mm diameter connector bars with a universal silver-based and cadmium-free solder; (3) Results: The distractor described can be a useful tool to correct mandibular retrognathia and is better tolerated by patients, especially in severe cases; (4) Conclusions: The dental-anchored distractor increases the anterior mandibular bone segment without affecting the gonial angle or transverse angulation of the segments and avoids posterior mandibular rotation, overcoming the limitations of conventional surgical treatment.

## 1. Introduction

Mandibular hypoplasia is the most common growth disorder of the facial skeleton in Western Europe. With a prevalence ranging from 41% to 56%, this condition is commonly associated with an Angle class II occlusion, convex profile, and mandibular deficiency [1,2].

Mandibular deficiency has a variety of causes, ranging from embryogenesis to various postnatal causes. This condition can occur isolated or in association with congenital malformation syndromes and, depending on its severity, can require orthodontic-surgical-orthognathic treatment [2].

Several studies have reported that certain malocclusions, including skeletal Class II, are associated with decreased masticatory efficiency, abnormal speech, pain, and decreased social interaction [3]. Bernabé et al. [4] conducted a study in Brazilian adolescents describing the impacts of oral health on the quality of life based on malocclusion. The authors verified that those with Class II malocclusion reported a higher direct impact on quality of life when compared with normal and Class I malocclusion.

The surgical and orthodontic treatment of mandibular deficiency progressed significantly in the second half of the twentieth century with the introduction of new techniques and materials. These procedures have the primary goal of restoring dental occlusion, masticatory function, respiratory function, and harmony in patients with class II skeletal malformations [5,6].

For the correction of this deformity, the most frequent treatment encompasses a conventional orthodontic-surgical-orthognathic protocol which is divided into four stages. The first is the presurgical orthodontic phase. This step is crucial as it is set to eliminate tooth compensation, harmonize the dental arches, and place the teeth in a stable position regarding the bone bases. This is followed by the surgical phase comprised of a bilateral sagittal split osteotomy (BSSO) which is the most commonly performed technique (although it has several different variations). The post-surgical stage is a combination of recovery and orthodontic refinements. The final stage is an optional surgical intervention to remove the osteosynthesis plates.

The surgery itself is performed under general anesthesia and usually requires four to six weeks of rigid or non-rigid maxillomandibular fixation. As for the post-surgical orthodontic phase, it takes around six months on average. During this period, bone tissue is consolidated and the dental occlusion is adjusted in an attempt to prevent or camouflage small skeletal relapse.

Although this protocol is very commonplace, it is not exempt of risk or handicaps. This intervention will generally increase the overall treatment time. As mandibular premolar extractions are usually required in order to allow for dental compensation correction, the surgery is only recommended to be performed on adult patients (after completion of skeletal growth). It also presents itself with a high relapse rate and is an expensive and temporarily incapacitating procedure [7,8]. To overcome these limitations, conventional surgical treatment is gradually being replaced by other techniques such as mandibular distraction osteogenesis (DO) [9].

The American Association of Oral and Maxillofacial Surgeons and other researchers have reported several situations for which distraction osteogenesis should the chosen procedure for mandibular elongation. These include hemifacial microsomia (i.e., unilateral distraction of the ascending ramus, mandibular angle, or posterior part of the mandibular body); mandibular body segmental defects due to trauma or tumor entity; class II syndrome caused by mandibular position (retrognathia) or dimension insufficiency (brachygnathia); micrognathia caused by trauma and temporomandibular joint ankylosis; vertical alveolar distraction for occlusal plane correction and deficiencies in the position and dimension of the alveolar ridges for dental implant rehabilitation; and for mandibular elongation in obstructive apnea syndrome [10,11,12,13,14,15,16].

Several authors have attempted to compare BSSO and DO procedures. However, the reported results were contradictory. Al-Moraissi et al. [1] conducted a systematic review with a meta-analysis to determine whether there were differences in skeletal stability and neurosensory disturbance of the inferior alveolar nerve between BSSO and DO concerning mandibular advancement surgery. A statistically significant difference in neurosensory disturbance of inferior alveolar ramus function was found between the BSSO and DO (*p* = 0.004), and the authors’ findings showed that distraction osteogenesis significantly reduced the incidence of neurosensory disturbance of the inferior alveolar ramus after lengthening of the retrognathic mandible when compared to BBSO. However, these findings could not be confirmed in other studies. Akkerman et al. [17] conducted a review comparing both techniques for mandibular advancement since it was assumed that DO would result in better stability and lower neurosensory disturbances of the inferior alveolar ramus. Nonetheless, based on the included prospective studies, the authors concluded that BSSO is not only superior in terms of stability and neurosensory disturbances in the inferior alveolar nerve but that it also seems to result in less pain and lower total costs. Some systematic reviews that compared the effectiveness of distraction osteogenesis versus orthognathic surgery in cleft patients verified that both methods can produce significant hard and soft tissue improvements but that the relapse rate is lower in the distraction osteogenesis group five years post-surgery [13,18].

The available experimental and clinical studies mostly concern external distractors. Despite their biomechanical advantages, external distractors have more side effects from the patients’ point of view. As it is more disabling, psychosocial problems can arise due to the distractor’s visibility and volume. There is also perceptible scarring on the face. The disadvantages do not end there, as there is instability in distractor fixation and a risk of dental and/or nerve injury (placement-osteotomy-removal); edema is generally present, and the wounds are propitious to localized infections [19,20,21,22,23].

All of these limitations often resulted in the rejection of the external distractors, requiring the development of alternative mechanics. The intraoral distractors that were nothing more than miniaturizations of existing external devices and/or adaptations of orthodontic devices for maxillary disjunction.

McCarthy et al. [24] in 1992 were the first to use internal distractors and were able to eliminate the problems of scarring sequelae and the visibility of external distractors, but the risks and limitations of fixation remained.

Regarding dental-anchored distractors, Guerrero used an intraoral distractor to correct mandibular deformities for the first time in 1990, using a modified Hyrax-type appliance cemented to the first premolars and lower molars [20]. Razdolky et al. [25] pointed out several benefits of these devices over traditional distractors (namely fixation, as it was performed directly on the teeth and no surgical intervention was required for placement or removal, making the internal dental-anchored distractor less invasive) [23]. The surgical act can be reduced to only cortectomies or osteotomies, allowing for an outpatient procedure without the need for an extensive stay in the hospital or general anesthesia. When compared to traditional distractors the device placement is easier allowing for the distraction to occur parallel to the chosen vector or occlusal plane, avoiding posterior mandibular rotation, and making orthodontic treatment easier [26].

The aim of this study is to describe a new custom-manufactured dental-anchored distractor anchored in the first molar and lower canine to perform the sagittal distraction of the mandible as parallel to the occlusal plane as possible.

## 2. Materials and Methods

**Figure 1:**
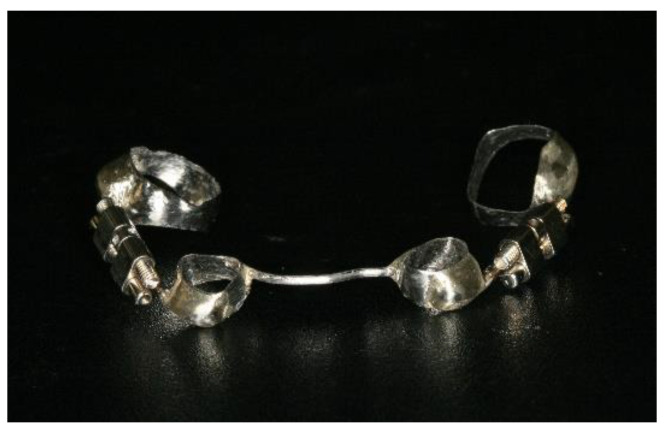
Photography of the custom-manufactured dental-anchored distractor.

A new custom-manufactured dental-anchored distractor design was elaborated and anchored in the firs molar and lower canine. It consists of a stainless-steel disjunction screw, adapted, and welded to the orthodontic bands through two 1.2 mm diameter connector bars, with universal silver-based and cadmium-free solder, 0.1 mm in diameter (Figure 1). The mechanism of the screw is the rotation-pressure type, allowing the dismultiplication of the effort for the elongation, with a maximum expansion of 12 mm. The rotational movement of 360° around its axis produces a translational movement along this axis (sagittal elongation) of 0.8 mm. A single screw activation allows only a rotation of 90° (1/4 turn), producing a 0.2 mm elongation, and features a brake system that prevents the accidental return of the activation.

Surgically, the cortectomy is performed between the lower premolars, with slight deviation to preserve the continuity of the inferior alveolar neurovascular bundle.

## 3. Results

**Figure 2:**
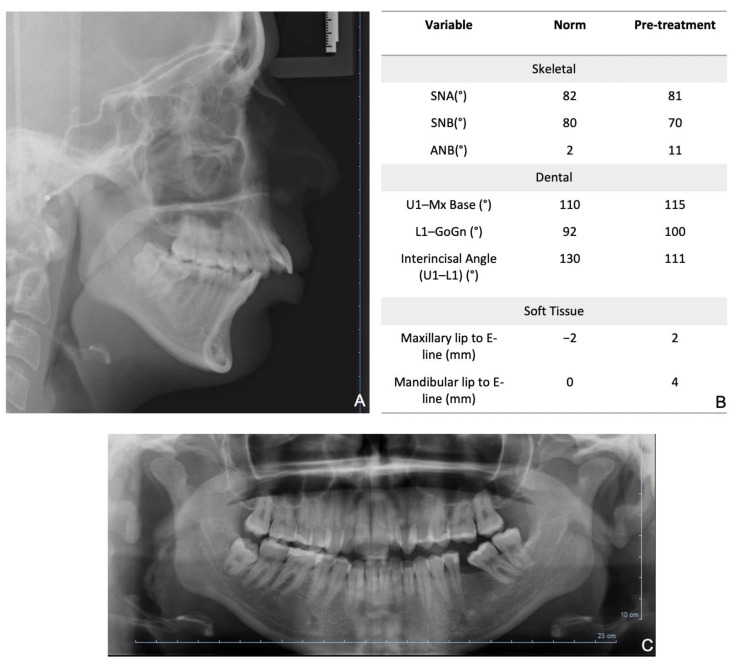
Pretreatment records: (**A**) initial ortopantomography: (**B**) lateral cephalogram; (**C**) initial cephalometric measurement.

A 21-year-old female presented a skeletal and angle Class II malocclusion, a deviation from the dental midline, and a crowding in the lower arch. Figure 2 shows the pretreatment records.

**Figure 3:**
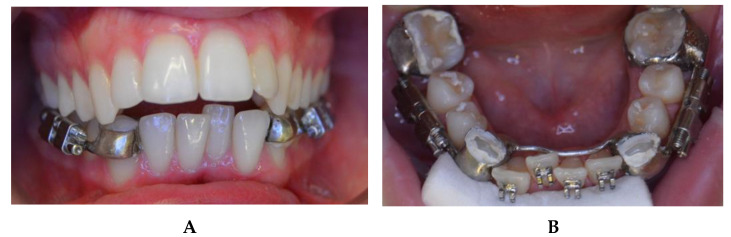
Intra-oral photographs: (**A**) frontal photograph before placement of the fixed multibracket appliance and surgery; (**B**) cementation of the distractor before surgery with Ketac™ Cem (3M-ESPE, Seefeld, Germany).

Initially, both arches were aligned and leveled with fixed orthodontic appliances (Roth 0.018—equilibrium^®^2 Dentaurum, Turnstrabe 31, Ispringen, Germany) for six months in order to provide enough interdental space to perform the osteotomies without damaging the adjacent teeth. A dental-anchored distractor was placed and the surgery was performed under general anesthesia with nasoendotracheal intubation (Figure 3). A buccal incision subperiosteal was made in order to expose the alveolar, basilar, and external face of the mandibular body. The bilateral cortectomies were performed vertically between the mandibular first and second premolar from the lower edge of the mandible towards the alveolar crest, and transversely to the mandibular body without perforating the lingual cortical bone, preventing the damage of lingual nerves. The lingual cortical bone was separated with an osteotome and, subsequently, bone mobility was checked.

**Figure 4:**
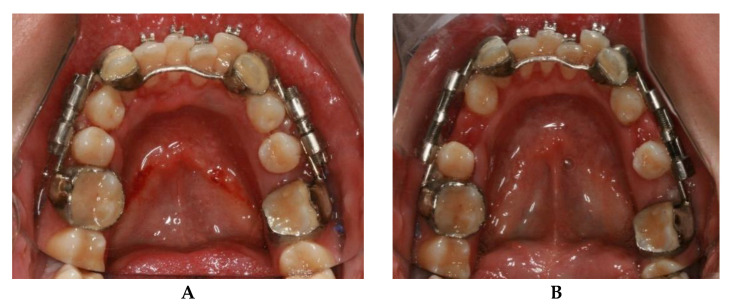
Intra oral mandibular photography before (**A**) and after the distraction osteogenesis (**B**) with dento-anchored distractor.

After seven days of latency, the process of increasing the mandibular length initiates, with a distraction rate of 1 mm/day (0.5 mm every 12 h). The distractors anchored to the teeth and parallel to the occlusal plane allow for a significant increase of the anterior bone segment without any side effects at the gonial angle or alteration of the transverse angulation of the bone segments (Figure 4). Therefore, the tooth-borne distractor produces a correct lengthening direction which facilitates the creation of the desirable stress force necessary for the osteogenesis process instead of an undesirable compression force.

**Figure 5:**
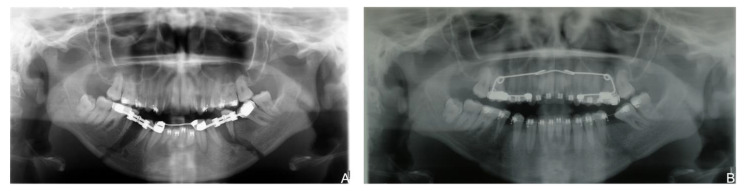
Orthopantomography immediately after the active period of distraction (**A**) and with six months consolidation period (**B**).

**Figure 6:**
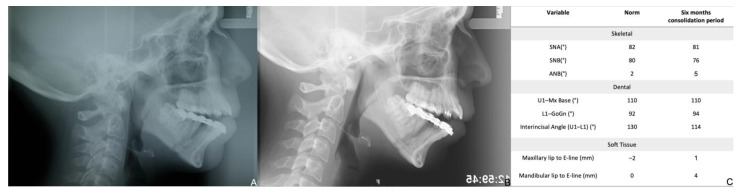
Post-surgical records: lateral cephalogram immediately after the active period of distraction (**A**) and with six months consolidation period (**B**); (**C**) cephalometric measurement with six months consolidation period.

During the active period of distraction (14 days), a mechanical force is applied to the soft callus, preventing fracture healing and creating a unique and highly dynamic microenvironment. The soft tissues, such as the epidermis, dermis, blood vessels, tendons, muscles, and nerves, gradually follow bone growth as a result of the tension forces applied to the bone segments (Figure 5 and Figure 6) [24,27,28]. The removal of the distractor was carried out after radiographic evidence of bone callus formation.

## 4. Discussion

Due to the enormous potential of distraction osteogenesis in the treatment of dentofacial deformities, the interest and use of this technique in orthodontics and maxillofacial surgery has grown significantly in recent years. Its late appearance in orthopedics can be explained in part by the anatomical characteristics of the craniofacial region, which make distraction devices difficult to apply. Indeed, the morphology of the bones of the facial mass is more complex, the scars are less tolerable than in other parts of the body, and conventional maxillofacial surgery techniques allowed for the resolution of a large portion of the severe facial deformations.

The main interest of distraction osteogenesis lies in its regenerative nature. The gradual and controlled elongation that allows tissue regeneration and repair occurs not only in the bone skeleton but also in soft tissue associated with it including the muscles, subcutaneous cellular tissue, and skin. This feature is particularly important in the treatment of dento-skeletal deformities that require mandibular advancement and in determining the choice of distraction osteogenesis technique over conventional orthognathic surgery, as well as substantially reducing the risk of relapse caused by strong soft tissue tensions [24,29,30,31]. Furthermore, this surgical technique can be used either in pediatric or adult patients [32,33].

Dental-anchored distractors are only used intraorally and are bonded to the teeth. Despite having significant advantages, such as having little psychosocial impact on patients, reduced dimensions, no risk of infection, being less invasive and requiring no surgical intervention in placement and removal, and having less morbidity and greater stability in the anchorage, they have been poorly studied for use in sagittal dentoskeletal deformities and have been primarily used to treat transverse jaw deformities [20,34,35]. Variation in design of the distractor can produce different outcomes. The most described distractor designs were tooth-borne and hybrid devices, in which the anchorage was performed through stainless steel crowns or miniplates [25,34,35,36]. However, this design involvesr difficult laboratory manufacturing process. The suggested new custom-manufactured dental-anchored distractor utilizes a hyrax screw and orthodontic bands customized to the size of the teeth accessible to any laboratory, making the manufacturing method more accessible. Furthermore, the inclusion of a connector bar in the anterior segment that connects both mandibular right and left sides allowed for a significant increase of the anterior bone segment without affecting the transverse angulation of the bone segments. Nonetheless, the main challenge of the intraoral distractors is controlling the vector direction; when improperly managed it can result in asymmetry or posterior rotation of the mandible [37]. The treatment plan should consider molar and canine final position in order to plan presurgical orthodontics with fixed appliances, with or without temporary anchorage devices, in order to eliminate dental interferences that may contribute to an unstable occlusion which in turn can alter the distraction vector [38]. More recently, the introduction of 3D technologies can improve the planning of mandibular osteogenic distractions. 3D treatment plans provide the ability to test various treatment hypotheses by changing osteotomy location and distractor configurations, granting an immediate prediction of the patients’ profile during the post-surgical period. In order to achieve this, a custom cutting guide is manufactured and a predetermined distraction vector is implemented in the distractor angle guide [39,40].

## 5. Conclusions

The use of a dental-anchored distractor allows its placement and removal without demanding surgery. The osteogenic distraction using a dental-anchored distractor is efficient in the sagittal lengthening of the jaw. Therefore, this procedure represents a new orthodontic surgical treatment approach alternative to the mandibular bilateral sagittal split osteotomy and should be considered as a treatment option for mandibular hypoplasia.

## Data Availability

The data presented in this study are available on request from the corresponding author. The data are not publicly available due to ethical concerns.
